# Simplified follow-up after medical abortion using a low-sensitivity urinary pregnancy test and a pictorial instruction sheet in Rajasthan, India – study protocol and intervention adaptation of a randomised control trial

**DOI:** 10.1186/1472-6874-14-98

**Published:** 2014-08-15

**Authors:** Mandira Paul, Kirti Iyengar, Sharad Iyengar, Kristina Gemzell-Danielsson, Birgitta Essén, Marie Klingberg-Allvin

**Affiliations:** 1 Department of Women’s and Children’s Health, Karolinska Institutet, University Hospital, Stockholm, Sweden; 2 Department of Women’s and Children’s Health, IMCH, Uppsala University, Uppsala, Sweden; 3 Division of Reproductive Health at Action Research, Training for Health (ARTH) Society, Udaipur, India; 4 School of education, health and social studies, Dalarna University, Falun, Sweden

**Keywords:** Medical abortion, Simplified follow-up, Safe abortion, Acceptability, India

## Abstract

**Background:**

The World Health Organisation suggests that simplification of the medical abortion regime will contribute to an increased acceptability of medical abortion, among women as well as providers. It is expected that a home-based follow-up after a medical abortion will increase the willingness to opt for medical abortion as well as decrease the workload and service costs in the clinic.

**Methods/Design:**

This study protocol describes a study that is a randomised, controlled, non-superiority trial. Women screened to participate in the study are those with unwanted pregnancies and gestational ages equal to or less than nine weeks. The randomisation list will be generated using a computerized random number generator and opaque sealed envelopes with group allocation will be prepared. Randomization of the study participants will occur after the first clinical encounter with the doctor. Eligible women randomised to the home-based assessment group will use a low-sensitivity pregnancy test and a pictorial instruction sheet at home, while the women in the clinic follow-up group will return to the clinic for routine follow-up carried out by a doctor. The primary objective of the study this study protocol describes is to evaluate the efficacy of home-based assessment using a low-sensitivity pregnancy test and a pictorial instruction sheet 10–14 days after an early medical abortion. Providers or research assistants will not be blinded during outcome assessment. To ensure feasibility of the self-assessment intervention an adaption phase took place at the selected study sites before study initiation. This resulted in an optimized, tailor-made intervention and in the development of the pictorial instruction sheet with a guide on how to use the low-sensitivity pregnancy test and the danger signs after a medical abortion.

**Discussion:**

In this paper, we will describe the study protocol for a randomised control trial investigating the efficacy of simplified follow-up in terms of home-based assessment, 10–14 days after a medical abortion. Moreover, a description of the adaptation phase is included for a better understanding of the implementation of the intervention in a setting where literacy is low and the road-connections are poor.

**Trial registration:**

Clinicaltrials.gov NCT01827995. Registered 04 May 2013.

## Background

The maternal mortality ratio in India decreased from 570 in 1990 to 178 per 100,000 live births in 2010 [1,2]. Despite these remarkable gains, India remains responsible for 20% of maternal deaths globally [3]. Abortion represents 8-18% of maternal deaths in India today even though abortion is legal since the Medical Termination of Pregnancy (MTP) Act went into effect in April 1972 [4,5]. Unsafe abortion is defined by the World Health Organization (WHO) as a procedure for terminating an unintended pregnancy carried out either by persons lacking the necessary skills or in an environment that does not conform to minimal medical standards, or both [6].

According to the MTP Act, abortions are legal up to 20 weeks if the mother’s life is at risk, if the foetus suffers from severe abnormalities or, if the contraceptive method used, fails to prevent pregnancy. Governmental facilities may provide abortion care if employing a practitioner trained to provide abortions. Private clinics, on the other hand, need a governmental approval to be allowed to conduct abortions [7]. In spite of the existing legal framework in India, a literature review from 2002 suggests that only 15% of abortions are performed within this legal framework [8]. Apart from lack of access, this may be explained by women’s lack of awareness of the legal status of abortions and thus they opt for illegal providers. Concerns about confidentiality, cost and quality of available services are other reasons reported to affect women’s choice of abortion provider [9]. In 2002, the Drug Controller of India approved the use of mifepristone for the purpose of termination of pregnancy up to 49 days of gestation. In 2006, misoprostol was approved for treating gynaecological conditions, including early abortions [10]. In 2008, the Central drug standard control organisation approved the combipack (1 tablet of mifepristone 200 mg and 4 tablets of misoprostol 200 mcg) for medical abortion for up to 63 days of gestation, although the MTP Act still state 49 days as the limit for medical abortion [11]. Medical abortion is feasible and acceptable in India [12,13], however, implementation of medical abortion as an alternative to surgical abortion has been slow on a national scale. As of 2008 only 3% of qualified abortion providers reported to provide medical abortion, and 23% intended to do so in the future [14].

Medical abortion using a combination of mifepristone and the prostaglandin E1 analogue misoprostol is globally recognized as a safe and effective method for induced abortion [15,16]. Women’s acceptability of medical abortion is influenced by factors such as duration and amount of bleeding, experienced pain during the procedure and the number of clinical visits required [17]. Moreover women seem to prefer a medical, non invasive, method rather than a surgical method [18]. The standard procedure for medical abortion in India commonly requires three clinical visits carried out by a physician [11]. Existing WHO guidelines suggest that a medical follow-up is not necessary after an uncomplicated medical abortion. This is applicable for women with a gestational age up to 12 weeks, if the woman receives proper counselling at the time of her abortion. Although a clinical visit may not be required, follow-up is important to detect an on-going pregnancy [19]. The current practise of three clinical visits results in considerable service costs and misuse of resources. A number of studies establish the feasibility and safety of home-administration of misoprostol [20-23]. Recent studies, majority of which are survey studies with small study samples, evaluate alternative means of follow-up after a medical abortion [24-26]. Another study demonstrates that follow-up can be carried out using remote communication technologies [27]. Moreover, women conducting a low-sensitivity urinary pregnancy (LSUP) test two weeks post early medical abortion in combination with a telephone follow-up by a health provider has been implemented as standard procedure in a general hospital in Edinburgh [24,28]. Other studies proved feasibility of self-assessment with a high sensitivity pregnancy test at 30 days after abortion or a semi-quantitative pregnancy test post abortion, both combined with a telephone follow-up [25,26,29]. The above mentioned studies required the informants to own a phone, be literate, have access to proper infrastructure such as road connections and the presence of readily accessible emergency care and majority were carried out in high-income countries [25,26,30]. The absence of these conditions would raise a question of feasibility of simplified follow-up in a low-resource setting. The WHO request more evidence regarding self-assessment in their technical guideline for safe abortions [19].

## Methods/Design

### Aim and hypothesis

This randomised controlled trial (RCT) aims to evaluate the efficacy of home-based assessment after an early medical abortion as well as the acceptability and feasibility of the intervention in a low-resource setting. We hypothesize that home-based assessment after early medical abortion will be as effective as routine clinic follow-up and will increase women’s acceptance towards medical abortion. This study protocol describes the design and method of the RCT along with the course of adaptation and implementation of the study intervention.

### Participating institutions

The Department of Women’s and Children’s Health at Karolinska Institutet in Sweden; the Division of Reproductive Health at Action Research and Training for Health (ARTH) Society, Udaipur in India and the Department of Women’s and Children’s Health at Uppsala University in Sweden designed and developed the study collaboratively. The study follows CONSORT guidelines and is registered at Clinicaltrials.gov (No. NCT01827995).

### Ethical approval

The Institutional Ethics Committee of ARTH has given ethical approval of the study and the Indian Council of Medical Research has approved the participating institutions to carry out the study in India.

### Definitions

In this study, we define complete abortion as when no signs of pregnancy or retention of product of conception (POC) is present, or upon a negative LSUP test, and no further treatment is needed for termination of the pregnancy. We define incomplete abortion as partially retained POC, sometimes with an enlarged size of the uterus or a positive LSUP test. Incomplete abortion requires further treatment, preferably with additional misoprostol or manual vacuum aspiration (MVA). We define on-going pregnancy as persistent pregnancy symptoms, no POC expulsion and enlargement of the uterus [19]. Moreover, with the LSUP-test we are referring to the combined low- and high- sensitivity pregnancy test purchased from VEDA.LAB (http://www.vedalab.com). The high-sensitivity component is equal to a standard pregnancy test and has a sensitivity of 5 units/litre. The low-sensitivity component has a sensitivity of 1000 units/litre, and is suitable for the use to assess abortion outcome at 10–14 days post abortion. In this study, the low-sensitivity component is of interest, however since there are no separate LSUP tests available on the market we used the combined test. Moreover, the test used in this study is not available in India, however several international pregnancy test manufacturers are currently working to develop LSUP-tests, and its availability on the market is likely to increase in the near future.

### Study setting

ARTH, a not for profit public health organisation, operates three rural and one urban clinic in and around Udaipur in the southern part of Rajasthan state in India. ARTH’s three rural clinics cater to women living in an area characterized by poor road connections, poverty, and low literacy among women (48.5%). The women in this area belong to different social groups, where about half the population belong to the marginalized groups, scheduled caste or scheduled tribe. Apart from carrying out domestic work, women in this area are largely engaged in marginal farming, wage labour and animal husbandry. In contrast to the rural clinics, ARTH’s urban clinic caters to a wider range of women, however majority from a low socioeconomic stratum. The services were made available at the ARTH clinics at a cost to the use of about 4 USD equivalent. To increase the urban sample, we approached two private nursing homes known to provide medical abortion in the city of Udaipur to act as study sites. Their services, subsidized for the purpose of the study, range between 6.5 and 16 USD. In total six clinics, three urban and three rural clinics were included as study sites. Approximately 550 women receive MTP from ARTH’s health centres annually, and of these about 450 women opt for medical abortion. The summarized caseload of the private clinics is an estimated 100 medical abortions per year. Home administration of misoprostol accounts for about 40% of all medical abortions.

Inclusion criteria:

–Women with unwanted pregnancies, who opt for medical abortion at any of the six above described study sites.

–Gestational age of less than or equal to nine weeks, as assessed by an obstetrician.

–Residing within the identified study area consisting of 70 villages in a range of 25 km from the rural clinics (at the rural clinics only).

–Agree to follow-up after 12–15 days, by either phone or home-visit.

Exclusion criteria:

–Women with known contraindications to medical abortion, as assessed by the doctor

–Haemoglobin-value less than 8.5 g/l.

–Younger than 18 years

### Sample size

The success rate of medical abortion is 95% [31,32] resulting in a complication rate estimation of 5% in both groups. The primary outcome define the sample size estimation where the 5% non-inferiority margin (delta) establishes the non-inferiority of the home-based self-assessment following medical abortion as compared to the routine clinic follow-up. The delta margin is defined by the clinical assessment standpoint where a 5% difference does not have clinical importance. To proof the non-inferiority of the intervention at a significance level of α = 0,05 and a power of 80%, 596 women are needed. The lost to follow-up estimation of 20%, based on field-testing in the study area, resulted in a final sample size of 716 women. A two-sided 95% confidence interval (CI) (or a one-sided 97,5% CI) will determine the difference in percentage of women requiring surgical intervention from the two groups.

### Research team

In addition to the obstetricians and nurse-midwives already working in each of the study sites, four research assistants (RAs) were assigned to the project. The RAs are women with a bachelor’s degree and a few years of experience working in the study area. They speak the local dialect and are aware of the local traditions and customs. In addition, a research associate for coordination of the study and a data manager to monitor the data entry and to attend to data-entry queries were assigned.

The research team received training before the initiation of the study-related activities and a final orientation will be provided at the time of initiation of data collection. Providers will be included in the orientation at the time of study initiation to create a general on-site understanding of the study. A manual on how to carry out the study and fill in the questionnaire will be available at each study site.

### Adaptation phase

**Figure 1:**
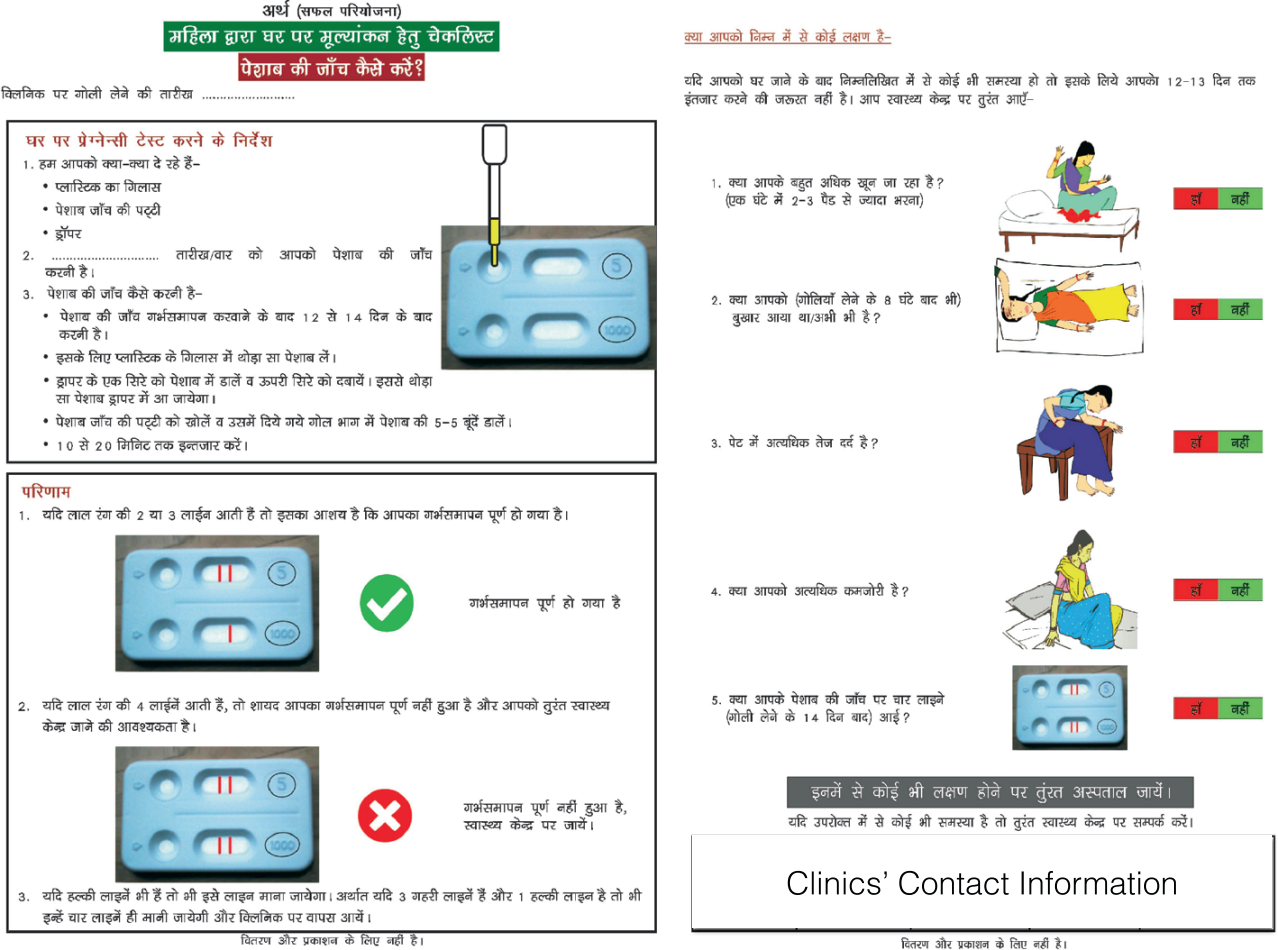
**The pictorial instruction sheet used for the home-based assessment group.** The instruction for use and interpretation of the LSUP-test (left side) and the pictorial explanation of the danger signs that may occur after a medical abortion indicating that the woman need to return to the clinic or consult a health care provider (right side). The pictorial instruction sheet consists of both pictures and text to cater to the different needs and literacy levels of the inhabitants in this setting.

We field-tested the intervention to ensure feasibility and adaptation to the context. This included the evaluation of the LSUP-test procedure and the pictorial instruction sheet explaining how to use the LSUP-test as well as possible danger signs of medical abortion (Figure 1). The Indian national standard guidelines for comprehensive abortion care and other similar pictorial guidelines used in the context of medical abortion [11,33] inspired the pictorial instruction sheet used for this study. Women from the designated field area interpreted and explained what they understood from the instruction sheet to validate the content. The pictorial instruction sheet was repeatedly modified and field-tested until it could fully aid women to understand how to use the LSUP-test by themselves, as well as when to return to the clinic in the event of any side effects or complications. While testing the intervention, we also tested and adapted the study questionnaire. This was to ensure a good flow in the questioning and an appropriate way of posting the questions. All questions were translated to the local language and the meaning of content was thoroughly discussed and concluded upon together with clinical staff and RAs. In addition, the obtaining of the informed consent was field-tested, as most participants cannot read the information sheet for themselves. This resulted in RA administered consent taking and a simplification of the text in the consent form, however in accordance with ethical committee guidelines.

The study procedures of the intervention group were field tested in the clinics of ARTH, during October and November 2012. We assigned all eligible women that agreed to participate to the home-based assessment intervention to carry out the LSUP-test (n = 33). At the time of follow-up the RAs interviewed the women regarding how much the woman had understood of the informed consent, the LSUP-test procedure and whether she felt safe about participating in the study. The responses from the women gave an understanding of the feasibility of the study protocol and the home-based assessment procedure as well as the accuracy of the questions in the questionnaire.

When assessing means of follow-up we found that majority of rural women required home-visits, largely due to the lack of personal ownership of cell-phones and hence confidentiality. To maintain the confidentiality of the women at the time of the home-visit the researchers adopted techniques such as camouflage visits to other women in the village, women of reproductive age commonly with recently born babies or women that were pregnant. In addition, in case any other family member or neighbour came in to the room of the interview, the RA immediately altered the topic of conversation. If complete privacy could not be obtained during the interview the RA would come back at another time, alternatively meet the woman elsewhere at her convenience e.g. by the water pump or in her farm. In the cases of a positive LSUP test or the presence of any danger signs, routes of referral and follow-up needed to be tested and streamlined. In addition to these adjustments, the routine of providing a contraceptive method at the time of follow-up needed alteration in the home-based assessment group since they no longer presented in the clinic. Discussions with the research staff about limiting loss of contraceptive uptake resulted in an updated clinic guideline where contraceptive counselling and provision, where applicable, was provided at the time of the first encounter or at the time of clinic administration of misoprostol.

Once the home-based assessment group was field tested, and the questionnaires and the pictorial instruction sheet finalized, we initiated a field test of the clinic follow-up procedure (n = 36). The clinic follow-up group followed the clinic protocol with two to three clinical visits in total. Instead of routine follow-up at seven days post abortion the follow-up occurred at 10–14 days post initiation of the medical abortion for the purpose of the study as well as to avoid over-diagnosis of incomplete abortions. Previous experience from service provision in this field area indicates that a large proportion of women do not return for clinic follow-up after an abortion. This affects the outcome as well as the validity of the study, which is why additional efforts were put into finding means of retaining the women in the study. Reasons why women would not return for the clinical follow-up were explored, and it was found that lack of transport and loss of income were major contributors to the drop-out. Provision of financial reimbursement for transport costs was tested and proved efficient in enhancing successful clinic follow-up. In addition, women mentioned lack of time as a reason for not returning to the clinic unless they suffered from any complications.

### Randomisation procedure

**Figure 2:**
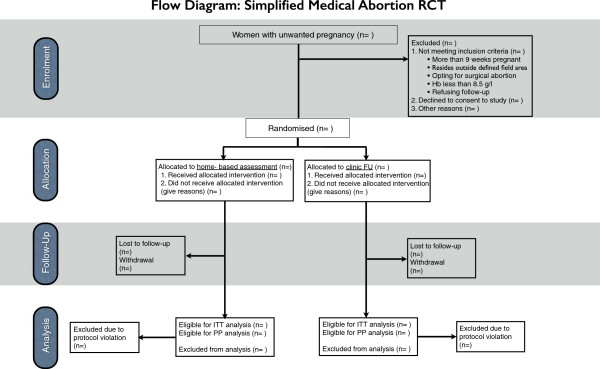
**Flow diagram of the study protocol.** A flow diagram developed according to CONSORT guidelines [37], describing the enrolment, randomisation, follow-up and analysis of the study.

The randomisation list will be generated using a computerized random number generator (random allocation software 2.0 [34]). The staff generating the list and preparing the opaque sealed envelopes are not to be involved in any data collection. The randomisation procedure is visualised in the flowchart (Figure 2). Randomization of the study participants will occur after the first clinical encounter with the doctor. Subsequently, the service provision will occur in a uniform and standard manner regardless of group allocation or study participation. Further blinding is not possible since the means of follow-up are depending on the group allocation. The clinical procedure on day one and three will remain the same regardless of group allocation of the woman. However, the location of misoprostol administration on day three, at home or in the clinic, is agreed upon based on the providers’ assessment together with the woman’s preference. Thus, this decision is made before the enrolment in the study. Block randomisation will be applied to ensure equal distribution of the two study groups in each clinic. Each clinic will be provided with envelopes according to their caseload. Consequently, the number of study participants will vary in each clinic.

Women who meet the inclusion criteria and who provide consent will be randomised into either home-based assessment (intervention) group or clinic follow-up (control) group. The RA at each clinic will carry out the randomization through picking the envelope with the lowest serial number available in the clinic. Each woman will be assigned a personal identification number once they consent to participate in the study. This number will then be used for the randomisation process as well as on every form of the questionnaire instead of the recording of names.

### Study protocol

#### All women - recruitment of women on day 1

After the doctor assesses the woman for eligibility of early medical abortion, the clinical eligibility of the woman to participate in the study will be assessed. Once this is established, the RA will assess the non-clinical eligibility of the woman including criteria such as age, area of residence or ownership of a phone, and whether the woman agrees to follow-up at home or in the clinic.

When a woman fulfils eligibility criteria, she will be provided with participant information, written or RA administered, and asked to give consent to participate in writing. Once the woman has agreed and signed the consent form, she will be randomised to home-based assessment group or clinic follow-up group. All women enrolled in the study will be registered in the trial registry, where personal information and means, location and date of follow-up will be recorded. In case a woman fails to be successfully contacted at the time of follow-up, the reason thereof will be noted in brief in the trial registry as well as further attempts to contact the woman. Women will receive instructions of the follow-up procedure, according to group allocation, on day one.

#### Misoprostol provision on day 3

A nurse or a doctor will supervise the administration of misoprostol to the women returning to the clinic on day three. Misoprostol will be provided and the woman is retained in the clinic for observation during one to five hours, depending on clinic, practitioner and woman’s availability. A clinical provider, commonly a nurse, will record any side effects and the abortion outcome upon discharge. Pelvic examination for confirmation of POC expulsion is not considered standard procedure.

##### Home-based assessment group

The intervention in this study consists of a home-based assessment carried out by the woman herself 10–14 days after an early medical abortion using an LSUP-test. The 10–14 day time frame is influenced by the sensitivity of the LSUP-test (1000 units/litre) [35]. It has been established that the human chorionic gonadotropin (hCG) levels of women with a gestational age no more than nine weeks drops substantially at 10–12 days after a medical abortion. However using a high-sensitivity pregnancy test or conducting the follow-up too early would result in increased numbers of false positives [36]. Waiting for four weeks to evaluate the abortion outcome may result in on-going pregnancies with gestation above 12 weeks. In this study setting, abortions are not as readily available after 12 weeks of gestation making an early detection of on-going pregnancy crucial. Women allocated to this group will receive an LSUP-test along with a pictorial instruction sheet (Figure 1). The women will be followed-up by an RA contacting the women over phone or through a home-visit, 12–15 days after initiation of the medical abortion. This is to ensure that the woman had enough time to carry out the LSUP-test by herself on day 10–14 before the contact with the RA. If the woman is not found at home or cannot be reached over phone, two additional attempts will be made to contact the woman.

#### Instructions for home-based assessment group

RAs will use a prototype of the LSUP-test to demonstrate how to use it along with the pictorial instruction sheet. Means of follow-up and date and time will be decided upon according to the woman’s preference. If the woman has a phone and is willing to receive a phone call, this will be the primary choice of follow-up. When women do not have access to a confidential phone, a home-visit will be suggested as an alternative. If the woman prefers neither they are advised to come back to the clinic for follow-up after having conducted the LSUP-test at home. These women will only meet with an RA at the time of follow-up – no clinical care will be offered unless the woman presents with complications or a positive LSUP-test, this will be recorded accordingly.

#### Follow up of home-based assessment group on day 12–15

At the time of follow-up, the RA will record the outcome of the LSUP-test along with side effects and other complications as reported by the woman. The women have been informed to return to the clinic in case of a positive LSUP test. However, the RA will refer the women that have not yet returned by the time of the follow-up visit to the clinic. To understand whether women are capable of assessing their abortion outcome using and interpreting the LSUP-test by themselves, different questions in the research questionnaire have been included. This is to distinguish whether the RA assisted the woman to conduct the LSUP-test at the time of follow-up or whether the woman had done it and interpreted it herself in advance of the RA consultation. In addition to the LSUP-test result the following set of questions will be asked by the RA to screen for other complications as well as the woman’s capacity to assess any side-effects: Do you think your abortion is complete? Did you see expulsion of products? If you had any pregnancy symptoms before are they gone now? Did you bleed more than twice as much as a normal period after taking the pill? Do you still have heavy bleeding? Do you still have severe abdominal pain? Do you feel sick? Have you had fever? Do you feel weaker than usual or like your whole body are aching? If the answer is yes to any of these questions, the RA may choose to refer the woman to the clinic regardless of the result of the LSUP-test. Important to notice is that the pictorial instruction sheet as well as the instructions given in the clinic emphasize the side effects or danger signs requiring a clinical visit. Hence the role of the RA is not to assess the abortion outcome of the woman during the follow-up visit. Moreover, women are not obliged to follow the advise of the RA to return to the clinic. Women with a negative LSUP-test, assessed as complete abortion at the time of follow-up, will be censored out of the study. Women that present with a positive LSUP test but do not return to the clinic will be approached through phone calls or home visits to confirm their abortion outcome in retrospect. Clinical records will be monitored in case of any adverse events or additional procedures in relation to the abortion for up to one-month post abortion.

##### Clinic follow-up group

Women in the clinic follow-up group will be encouraged to return to the clinic for a routine clinical examination by a doctor, 10–14 days after initiation of the medical abortion. In this study, women returning to the clinic will also be assessed with an LSUP-test carried out by clinical staff, and the same set of screening questions as stated above are asked to record any side-effects.

#### Follow-up of clinic follow-up group on day 10–14

Women allocated to the clinic follow-up group will be treated according to the routine practice of each clinic. For the cause of the study the time of the follow-up visit has been standardized to 14 days after initiation of medical abortion, or within the range of 10–14 days. At the follow-up visit, a doctor will assess the outcome of the abortion including a pelvic examination if regarded as necessary. Subsequently, an RA will carry out the final interview in the clinic before the woman will be censored out of the study. Women returning to the clinic will be reimbursed for their travel expenses (INR 200), while women that do not return for clinical follow-up will be reminded to return to the clinic over phone, where possible.

All women

#### Additional procedures, extra visits or lost to follow-up

Clinical staff will record any additional procedures (MVA, repeat misoprostol etc.) provided during the follow-up visit or at the time of misoprostol administration in the clinic, in the research questionnaire. For women returning before their scheduled follow-up with complications or adverse events, a doctor or a nurse will provide appropriate services. This will be recorded in an interim visit form as well as the clinical records of each study site. In addition, women will be asked at the time of follow-up, whether she had any interim visits or additional contacts with any clinic in relation to her abortion. Hence additional treatment or procedures will be recorded in the follow-up questionnaire as per the woman’s report. This is to register all additional procedures or visits of the women, since not all women may return to one of the study sites for interim visits. However, where possible and in the cases of surgical intervention the clinical reports kept by the women will be reviewed to distinguish the abortion outcome of the woman as well as confirm the procedure reported by the woman. Women where no clinical records are available and that are lost to follow-up will be approached through phone-calls, where applicable, or home visits during outreach activities in the villages within three months of their abortion, to confirm the abortion outcome.

### Ethical considerations

This study addresses abortion, commonly considered a sensitive topic due to the context and the social stigma. Therefore, extra measures to maintain the safety and confidentiality of the study participants are necessary. To address this we have developed a confidentiality protocol for each encounter with the women. At the time of enrolment, the women will be explained the study thoroughly and an informed consent will be taken from each woman, administered by the RA. The procedure of consent taking is carried out in a private setting, where none other than the woman and the RA are present, unless the woman is illiterate and a witness is required where this will be solved according to the woman’s preference of witness. Women, allocated to the home-based assessment group, will be able to choose means of follow-up appropriate to their personal situation: phone, home or clinic. At the time of follow-up of the home-based assessment group, two additional households will be visited and camouflage-interviews with young women will be conducted. This is to maintain confidentiality of the women and reduce suspicion from their neighbours. If, at any time during follow-up or interview with the study participants, another person interrupts the interview, the topic will be changed immediately to not reveal the purpose of the visit. If complete privacy cannot be maintained at the time of follow-up, the RA will come back at another time or meet the woman elsewhere at her convenience. If the woman prefers to discontinue the study due to privacy issues, the RA will not try to conduct the follow-up interview. The woman is allowed to exit the study at any time without giving a reason for doing so. These efforts are explained to the woman at the time of enrolment to avoid confusion at the event of topic change or camouflage interviewing.

### Measures

#### Socio-demographic and reproductive background data

Measures such as age, marital status, education level, caste and residential area will compile the socio-demographic information about the study participants. Moreover, the personal reproductive history will be recorded as number of births, number of living children, previous abortions, caesarean section and contraceptive use. Haemoglobin and the gestation will also be measured and recorded.

### Primary study outcome

*The primary outcome* of the study is the rate of complete abortion, when no signs of pregnancy or retention of POC is present, or upon a negative LSUP test, and no further treatment is needed for termination of the pregnancy, in both groups. Established classification of efficacy for medical abortion will be followed to determine the outcome of the abortion procedure [22,35]. Hence the primary outcome is; efficacy of home-based assessment, measured as the rate of complete abortion, relative to routine follow up at two weeks after initiation of medical abortion. The failed abortion rate will be measured through number of surgical interventions, clinical administration of additional misoprostol or on-going pregnancies established upon clinical examination.

### Secondary study outcome

*The secondary outcomes* include acceptability of the home-based assessment and the home use of misoprostol, when applicable, measured at the time of follow-up. To evaluate acceptability, we will ask women of their choice of method and means of follow-up in the event of a future abortion. Additional acceptability questions will regard women’s advice to a sister or a friend in need of an abortion. Finally, we will ask women with previous abortion experience to compare their previous experience with their current experience. For the purpose of efficacy of the intervention, we will evaluate recordings of the number of, and reasons for interim visits related to the medical abortion as well as side effects including infections, bleeding and pain. In addition, we aim to evaluate contraceptive uptake at 10–15 days after medical abortion along with previous use of contraception. Finally, the time consumed while travelling to and waiting in the clinic will be assessed as reported by the woman at the time of follow-up.

### Data management

Research assistants will send the completed research questionnaires from the field to the research office, where the research associate will code the forms. In case of any discrepancies or missing values, the forms will be returned to the field for correction. A data entry operator will enter the coded forms. No personal information of the study subjects will be entered in the database. Two different people will carry out double data entry of the forms at two different time points using a data entry format based on MS SQL database, specifically developed for the study. A query program has been developed and tested to detect any logical or range-related queries. The data manager will clean the data by resolving all queries on a monthly basis as well as an overall query run of the whole data set once all data has been entered.

### Data analysis

SPSS 20.0 will be used for statistical analysis. All analyses will be by intention to treat (ITT) and some will be analysed using per protocol analysis in addition to ITT. The non-inferiority hypothesis will be tested at a significance level of 5% (α = 0,05). Categorical outcomes will be presented using descriptive statistics and compared using χ^2^-test. Numerical variables will be assessed for significance using the student’s t-test. Intergroup comparison will be made between the clinic follow-up group and the home-based assessment group. Differences between groups will be analysed using relative risks (95% CI) and logistic regression for the relative differences. P-values equal to or lower than 0.05 will be considered statistically significant. Acceptability measures will be analysed descriptively, using numbers and percentages of women in the different categories.

### Quality control

Monthly meetings with research staff will be held to monitor and evaluate the study process. The research associate, using a checklist developed for the purpose, will carry out quality assessments on a regular basis and report them monthly. The quality control checklist includes aspects from all different components of the study protocol: (i) stock of medical abortion pills, pregnancy tests, envelopes etc., (ii) the completeness of registers, (iii) the recruitment process including the taking of informed consent, (iv) confidentiality, (v) follow-up procedure and (vi) the completeness of the questionnaires. This is to ensure the equal quality in all study sites as well as maintained quality throughout the study. In addition, an external monitoring board with experienced researchers have been assigned to evaluate the quality of the data at appropriate times; this is, according to CONSORT guidelines [37].

## Discussion

This paper summarizes the implementation and administration of an RCT that seek to test the feasibility and efficacy of home-based assessment after an early medical abortion using an LSUP-test and a pictorial instruction sheet. The intervention is designed to accommodate disadvantaged women living in remote as well as urban areas of India. This paper describes the study protocol of an RCT along with the course of adaptation and implementation of the study-intervention.

To increase generalizability of the results, exclusion criteria are limited to those of clinical importance to maintain the safety of the women. Due to the study design, women attending the rural clinics that are residing outside the defined study area will be considered ineligible. However, the study area includes 70 villages, where each village hosts approximately 10.000 inhabitants, including women from different socio-economic strata and with different educational backgrounds. Moreover, no area restrictions are applied to the women recruited in the urban clinics. Thus the sample will be generalizable. The sample size calculation is based on the likelihood of an early medical abortion resulting in a complete abortion (95%), rather than the likelihood of an on-going pregnancy (1-2%). This may limit the ability to identify the clinically important finding of on-going pregnancy. However, it is the commonly used outcome for trials investigating efficacy and safety of medical abortion due to its high success rate. This has been done similarly in trials by the WHO and Sunde-Oppegard et al. investigating schemes of follow-up after medical abortion [35]. With the additional assumption of a loss to follow-up of 20% based on the field testing, the total study sample required is 716 women, where 298 women with evaluated end point in each group is required to maintain power.

All recruited RAs have been working in the field under ARTH for several years, in their designated field area. This is considered a benefit, as it will diminish suspicion from neighbours during home visits, ensuring confidentiality of the women in the study.

Wolff argues that to conduct a service effectiveness study it is crucial that the intervention is accurately implemented and consistently operationalized throughout the study [38]. In addition, this needs to be recorded and described in detail so that it can be replicated elsewhere [39]. This is why adaptation of the study was of major importance as the intervention was to be implemented in a complex setting [39]. The rural areas, where the study will be carried out, have poor road connections and women have to travel long distances to access quality health care [40]. During the field-testing, the follow-up rates in the clinic follow-up group were lower than the follow-up rate in the home-based assessment group. This was resolved by providing an incentive for travel costs at the time of return to the clinic. Hence, the incentives increased the follow-up rates in the clinic follow-up group, although enough for the purpose of the study not as much as the follow-up rate of the home-based assessment group. This may indicate that women are reluctant to return to the clinic for follow-up unless they have complications. Home-based assessment may therefore accommodate the needs of the women, and hence facilitate health care seeking among women with failed abortions. It is believed that the acceptability of medical abortion increases when the number of required clinical visits decreases [17].

The research team consists of researchers from both India and Sweden. The researchers from India have experience from conducting research and interventions at a community level and the researchers from Sweden have experience in similar research in high-resource as well as other low-resource settings. Together, we developed the study tools considering the course of intervention as well as the contextual factors. Another strength of this study is the adaptation phase where, throughout the process of implementing and adapting the intervention, qualitative evaluations were carried out with both women and staff. These evaluations contributed to the adaptation of the intervention and indicated that women are capable of carrying out and interpreting an LSUP-test, making the study possible to conduct. The staff carrying out the study activities were continuously involved and consulted throughout the adaptation phase. This was considered important to optimise adaptation and facilitate implementation of the intervention.

Research assistants cannot be blinded at the time of randomisation, since different instructions are to be given to the women according to their group allocation. The lack of blinding is regarded a limitation in the study design. Important to notice is that the health care providers remain blinded at the initial encounter when the woman is assessed for eligibility of medical abortion as well as when location of misoprostol administration is decided. However, RAs are not blinded when administering the follow-up acceptability interview. Due to poor literacy among the majority of women in the study the acceptability questions cannot be self-administered. However, the follow-up acceptability interviews will be conducted in a confidential setting where no service providers are present. This is to avoid potential bias in the women’s responses regarding acceptability.

It is important to consider that the women in the home-based assessment group are aware that they will be followed-up at 12–15 days after their medical abortion. This may influence their care-seeking behaviour in response to the LSUP-test result as well as their acceptability of the intervention. The study essentially aims to evaluate a self-assessment regime, however due to the need of follow-up, whether home visit or phone call, in the study design the intervention is not a complete self-assessment, rather a home-based assessment followed up by a non-clinical provider. Although the role of the RA is not to assess the abortion outcome of the woman, she will refer the woman to the clinic if she feels any reason to do so. To further evaluate the feasibility of self-assessment, operational research is needed.

The women that opt for clinic use of misoprostol are retained in the clinic for one to five hours after administration. Expulsion of POC is not a necessity for discharge and regardless of whether the woman expel the POC or not, she is encouraged to assess the abortion outcome according to her study group allocation and will be followed-up accordingly. The women, whose complete expulsion of POC is confirmed by a clinician on day three, may be more informed of their abortion outcome and may therefore be unmotivated to carry out the LSUP test or return to the clinic for further follow-up. The primary reason for follow-up is to ensure complete abortion, if a clinician confirms complete POC expulsion already on day three, the follow-up may no longer be as relevant. However, assessment of POC is not clinical routine. Expulsion of POC will be recorded in the research questionnaire and can therefore be evaluated at the time of analysis. If expulsion of POC is confirmed in many cases it may affect the power of the study. Moreover, potential limitations of this study may be the loss of follow-up, if the women are not found at home or if successful contact could not be established in the home-based assessment group. More importantly if the travel reimbursement for the women in the clinic follow-up group does not overcome the loss of follow-up as thought, there may be a large dropout creating a skewed sample with underrepresentation from the clinic follow-up group.

## Abbreviations

ARTH: Action Research & Training for Health; CI: Confidence interval; hCG: human Chorionic Gonadotropin; ITT: Intention to treat; LSUP: Low-sensitivity urinary pregnancy; MVA: Manual vacuum aspiration; MTP: Medical termination of pregnancy; POC: Products of conception; RA: Research Assistant; RCT: Randomised controlled trial; SIDA: Swedish International Development Cooperation Agency; WHO: World Health Organization.

## Competing interests

There are not competing financial or non-financial interests in this study.

## Authors’ contributions

MP and KI participated in the implementation of the study including the adaptation phase as well as are involved in the training of the research staff and health providers. SI participated in conceptualising and implementing the study. KGD participated in conceptualising the study and developing the protocol. BE participated in developing the protocol and design of the study. MKA participated in conceptualising the study, developing the protocol and supervised implementation of the study including the adaptation phase. MP drafted the manuscript with all authors contributing to the manuscript. All authors approved the final manuscript.

## Pre-publication history

The pre-publication history for this paper can be accessed here:

http://www.biomedcentral.com/1472-6874/14/98/prepub
